# Long-Term Neurodevelopmental Outcomes After Hypoxic-Ischemic Encephalopathy Treated With Therapeutic Hypothermia: A Systematic Review

**DOI:** 10.7759/cureus.113597

**Published:** 2026-07-29

**Authors:** Maha Naif Alshreef, Majed Khidhran Alzahrani, Attia Shaban Attia Awad

**Affiliations:** 1 Emergency Medicine, Alhada Armed Forces Hospital, Taif, SAU

**Keywords:** hypoxic-ischaemic encephalopathy, long-term follow-up, neonatal encephalopathy, neurodevelopmental outcomes, therapeutic hypothermia

## Abstract

Therapeutic hypothermia (TH) is the cornerstone of treatment for infants with moderate-to-severe hypoxic-ischemic encephalopathy (HIE). The long-term neurodevelopmental outcomes after 18 to 24 months and the persistence or onset of impairments throughout later childhood, however, are still up for discussion. This systematic review evaluates the long-term neurodevelopmental outcomes (≥18 months) of babies with moderate-to-severe HIE treated with TH. A comprehensive literature search of PubMed/Medical Literature Analysis and Retrieval System Online (MEDLINE), Scopus, Web of Science, and Cochrane Controlled Register of Trials (CENTRAL) yielded 213 records. Following duplicate removal and screening, 10 studies encompassing 1,134 neonates met eligibility criteria. Two reviewers independently extracted the data and performed a design-specific risk of bias assessment. Methodological quality was evaluated using the Joanna Briggs Institute (JBI) Critical Appraisal Checklist for cohort studies, the Quality in Prognosis Studies (QUIPS) tool for prognostic factor studies, and the Cochrane Risk of Bias 2 (RoB 2) tool for randomized trials. Across the included studies, 52%-81% of survivors treated with therapeutic hypothermia achieved normal or mildly abnormal neurodevelopment at 18-36 months, with mortality ranging from 3.8% to 19.6%. Consistent predictors of adverse outcomes included delayed cooling, elevated lactate, severe EEG abnormalities, rewarming seizures, abnormal MRI, and prolonged birth depression. Novel biomarkers showed prognostic promise but require validation. Outcomes in low- and middle-income countries (LMICs) were variable, with high attrition limiting generalisability, and evidence suggested that impairments may emerge at school age, supporting surveillance beyond 24 months. TH is associated with positive long-term outcomes for the majority of survivors with moderate-to-severe HIE. However, neurodevelopmental impairments may emerge or persist into school age, necessitating surveillance beyond 24 months. While TH is effective in high-income settings, its benefits in LMICs depend on adequate resources and follow-up infrastructure. Validation of novel biomarkers and standardization of outcome measures are priorities for future research.

## Introduction and background

Among term and late-preterm newborns worldwide, one of the leading causes of mortality and long-term neurodevelopmental disability is still prenatal hypoxic-ischemic encephalopathy (HIE) [1]. Excitotoxicity, oxidative stress, mitochondrial malfunction, and finally programmed cell death are among the biochemical processes that arise from the neonatal period's interruption of cerebral blood flow and oxygen supply [1, 2]. Despite improvements in obstetric and neonatal care, the incidence of HIE is estimated to be between 1.5 and 2.5 per 1000 live births in high-income countries (HICs); substantially greater rates are seen in low- and middle-income settings, where the sickness burden is largest [3, 4]. Untreated moderate-to-severe HIE has a particularly worrisome natural history: newborns with moderate encephalopathy have a 10% chance of dying, and survivors have a 30% chance of developing neurodevelopmental impairments such as cerebral palsy, cognitive impairment, and language delay; for those with severe HIE, mortality approaches 60%, and the majority of survivors experience significant lifelong handicaps [5].

While the short-term efficacy of therapeutic hypothermia (TH) at 18-24 months of age is well established, important questions remain regarding the longer-term trajectory of neurodevelopmental outcomes beyond the initial follow-up period and the persistence or emergence of cognitive, motor, and behavioral impairments into later childhood and adolescence [6]. A secondary analysis of the National Institute of Child Health and Human Development (NICHD) trial by Pappas and colleagues highlighted that cognitive impairment remains an important concern for survivors of HIE even after TH and that early developmental testing may have limited predictive value for later cognitive outcomes [6, 7]. Furthermore, contemporary cohort studies have reported that subtle deficits in executive function, attention, processing speed, and academic achievement may become apparent only when children reach school age, suggesting that the full neurodevelopmental consequences of perinatal HIE may not be fully captured by assessments performed at 18-24 months [7]. Furthermore, there is still active research and discussion. On TH's effectiveness in low- and middle-income countries (LMICs), where the bulk of HIE cases occur, and resources for intensive care and long-term follow-up may be scarce [6]. This systematic review evaluates long-term neurodevelopmental outcomes in neonates with moderate-to-severe HIE treated with TH, identifies key clinical, biochemical, neurophysiological, and imaging predictors of unfavorable outcomes, and synthesizes evidence on neurodevelopmental trajectories beyond 24 months of age.

## Review

Methodology

This systematic review was conducted in accordance with the PRISMA 2020 guidelines [8].

Review questions

Given the multiple objectives, three distinct review questions were pre‑specified. Prognosis: Among infants with moderate‑to‑severe HIE treated with TH, what are the neurodevelopmental outcomes (rates of normal development, mild delay, moderate‑severe disability, and mortality) at ≥18 months of age? Prognostic factors: Which neonatal clinical, biochemical, neurophysiological, or imaging factors independently predict adverse neurodevelopmental outcomes in this population? Treatment effect: Does TH improve outcomes compared with normothermia or usual care, and do outcomes vary by cooling method (whole‑body vs. selective head; active vs. passive) or by country income level (HICs vs. LMICs)? 

Search strategy

We systematically searched PubMed/Medical Literature Analysis and Retrieval System Online (MEDLINE), Scopus, Web of Science, and Cochrane Controlled Register of Trials (CENTRAL) over a five‑year period using MeSH terms and free‑text keywords including "hypoxic‑ischemic encephalopathy," "therapeutic hypothermia," and "neurodevelopmental outcomes". Limits included English language, human studies, and full‑text availability. Reference lists of included studies and relevant reviews were hand‑searched for additional records. The full search strategy is presented in Appendix A.

Study selection and eligibility

All records were imported into Rayyan (Rayyan Systems, Inc., Cambridge, MA, USA) [9] for duplicate removal and blinded screening by two independent reviewers, with disagreements resolved by consensus or a third reviewer. Eligibility criteria were defined separately for each question. For Q1 and Q2 (prognosis and prognostic factors), we included prospective or retrospective cohort studies of neonates (≥35 weeks) with moderate‑to‑severe HIE treated with hypothermia, reporting validated neurodevelopmental outcomes (Bayley, Griffiths, Ages and Stages Questionnaires (ASQ), or composite death/disability) at ≥18 months. For Q2, studies were required to report multivariable‑adjusted effect estimates (e.g., adjusted OR/RR). For Q3 (treatment effect): We included randomised controlled trials (RCTs) and controlled cohort studies with concurrent normothermic comparators. Single‑arm studies were excluded from Q3.

General exclusions applied across all questions: follow‑up <18 months;>30% loss to follow‑up without sensitivity analysis; failure to separate TH‑treated infants; case reports/series (<10 participants); conference abstracts; reviews; and studies focusing exclusively on mild HIE or lacking severity reporting.

Data extraction

Two reviewers independently extracted data using a standardised form, collecting question‑specific information: for Q1, outcome proportions and mortality; for Q2, adjusted effect estimates and covariates; for Q3, intervention details, comparator characteristics, and comparative outcome data. Missing data were recorded as "not mentioned," and authors were contacted when feasible.

Risk of bias assessment

To account for the heterogeneous study designs included in this review, we employed design‑specific risk‑of‑bias tools rather than applying a single uniform scale. For single‑arm descriptive or outcome cohorts reporting overall prognosis without comparators (Q1), we used the Joanna Briggs Institute (JBI) Critical Appraisal Checklist for Cohort Studies [10], focusing on sample representativeness, adequacy of follow‑up, and validity of outcome measurement. For prognostic‑factor studies examining multivariable‑adjusted predictors (Q2), we applied the Quality in Prognosis Studies (QUIPS) tool [11], which evaluates six domains: study participation, attrition, prognostic factor measurement, outcome measurement, confounding measurement and adjustment, and statistical analysis. For RCTs and secondary trial analyses evaluating treatment effects (Q3), we used the Cochrane Risk of Bias 2 (RoB 2) tool [12]. Critically, we did not calculate or report composite summary quality scores (e.g., total stars or percentages), as such aggregated scores can obscure domain‑specific weaknesses such as high attrition, inadequate confounding control, or selective analysis. Instead, we narratively described the risk of bias across relevant domains for each study, allowing us to interpret findings with appropriate caution and to weight evidence according to methodological robustness. All assessments were performed independently by two reviewers, with disagreements resolved through consensus.

Data synthesis

Due to substantial clinical and methodological heterogeneity across studies, including differences in study designs, populations, assessment instruments, outcome definitions, and follow-up durations, a formal meta-analysis was not performed. Instead, a structured narrative synthesis was conducted, organized according to the three pre‑specified review questions. Within each question, findings were further synthesized thematically as follows:

For Q1 (prognosis), outcomes were grouped by domain (cognitive, motor, behavioural, and mortality) and by assessment age (18-24 months vs. beyond 24 months). Within each domain, we described the pattern of findings across studies (e.g., consistency, direction, and variability) without calculating pooled proportions or ranges that might imply statistical comparability.

For Q2 (prognostic factors), predictors were categorized into four thematic clusters: clinical, biochemical, neurophysiological, and imaging markers. Within each cluster, we examined the strength of evidence based on multivariable-adjusted estimates, sample size, and study quality, emphasizing the consistency of associations rather than deriving a single summary effect.

For Q3 (treatment effect), comparative findings were synthesized by comparator type (RCTs vs. controlled cohort studies), cooling modality (whole-body vs. selective head; active vs. passive), and healthcare setting (HICs vs. LMICs). Effect estimates were described qualitatively, with attention to the direction and magnitude of effects reported in individual studies, while avoiding meta-analytic pooling.

Results

**Figure 1:**
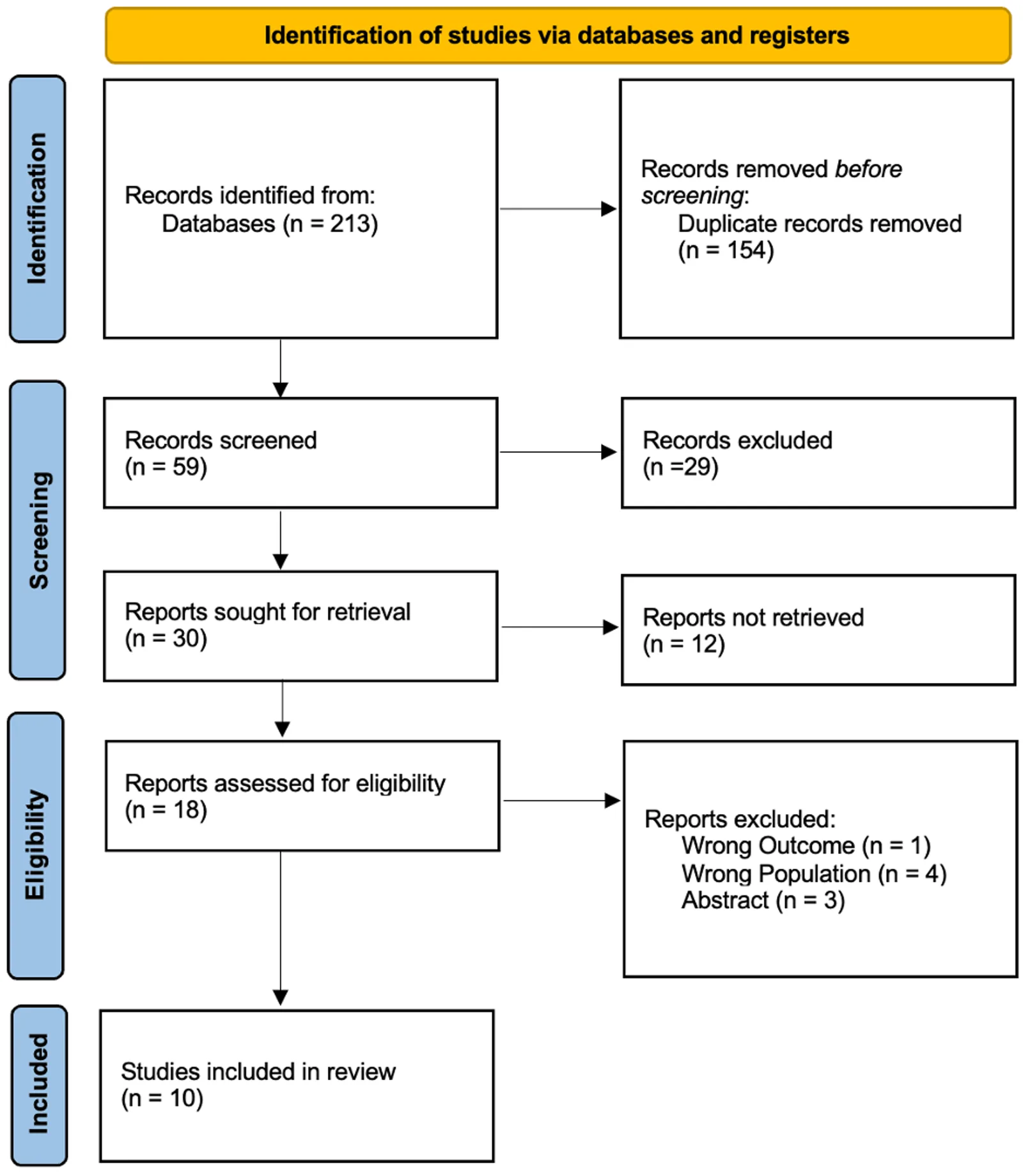
PRISMA flow diagram outlining the process of study selection

The research selection procedure for this systematic review is depicted in the PRISMA flow diagram (Figure 1). Database searches yielded 213 entries in total. Fifty-nine distinct records were screened after 154 duplicate records were eliminated. Thirty reports were sought for retrieval after 29 records were eliminated at the title and abstract screening stage. The remaining 18 full-text reports were evaluated for eligibility, but 12 reports were not accessible. Eight papers were eliminated after thorough review for the following reasons: incorrect population (n = 4), incorrect result (n = 1), and conference abstract without complete data (n = 3). As a result, after fulfilling all inclusion criteria, 10 publications were added to the final systematic review.

Table 1 shows that the included studies originated from diverse settings [13- 22]: three were conducted in the USA [17,21,21], one each in India [14], South Africa [15], Switzerland [18], and Italy [19], and one multicentre study from India, Sri Lanka, and Bangladesh [20]. Two studies did not specify the location [11,14]. Study designs varied considerably: four were prospective or observational cohorts [14,16,19,22], three were retrospective cohorts [15,18], one was descriptive observational [13], one was a nested cohort within an RCT [21], and one was an exploratory RCT analysis [20]. Glass et al. [17] was a cohort nested within an erythropoietin trial. Sample sizes ranged from 44 to 408 participants, with the largest being the HELIX trial analysis [20] (N=408) and the smallest being Jacob et al. [13] (N=44) and Giesinger et al. [16] (N=46).

**Table 1:** Demographics and study features of the included studies

Study (Author, Year (Reference))	Location	Study Design	Sample Size (N)	Sample Characteristics	Age at Follow-Up	Assessment Tool
Jacob et al., 2025 [13]	Not mentioned (NM)	Descriptive observational study	44	Outborn neonates with moderate-to-severe hypoxic-ischemic encephalopathy (HIE); 50% male; 86.4% birth weight >2.5 kg; seizures (88.6%), coagulopathy (72.7%)	36 months	Ages and Stages Questionnaire-3 (ASQ-3)
Veeraiah et al., 2025 [14]	India (Shivamogga)	Prospective study	47 (32 followed up)	Neonates born at or ≥36 weeks with moderate asphyxia-associated encephalopathy	18–24 months	The Bayley Scales of Infant and Toddler Development (BSID-IV) in its fourth version
Mbatha et al., 2021 [15]	South Africa (public hospital, Level 2 nursery)	Retrospective descriptive study	113 (received TH)	HIE: moderate 88%, severe 10%; neonates managed with therapeutic hypothermia (TH) outside the neonatal intensive care unit (NICU)	18–24 months	Griffiths Mental Developmental Scales (GMDS)/clinical disability assessment
Giesinger et al., 2022 [16]	NM (likely Canada)	Prospective physiological study	46 (43 with outcome)	TH is used to treat neonates with HIE who are older than 35 weeks.	Mean 18.9 months (±1.4)	The Bayley Scales of Infant and Toddler Development, Third Edition (BSID-III) is used to diagnose cerebral palsy.
Glass et al., 2023 [17]	USA (multicenter)	Cohort study (nested within a randomized controlled trial (RCT))	142	Hypothermia-treated newborns with HIE (engaged in an erythropoietin study)	2 years (24 months)	Gross Motor Function Classification System (GMFCS) and Bayley-III
Boerger et al., 2024 [18]	Switzerland (Zurich)	Retrospective cohort study	45	TH-treated neonates with mild and severe HIE	18–24 months	BSID-III
Ancona et al., 2025 [19]	Italy	Longitudinal study	53 (40 with long-term monitoring)	Neonates with HIE treated with TH	7 years	Intelligence quotient (IQ); cerebral palsy; death
Burgod et al., 2024 [20]	India, Sri Lanka, Bangladesh	Exploratory analysis of RCT (HELIX trial)	408	Treatment for infants with moderate or severe HIE who have whole-body hypothermia	18 months	Composite outcome: moderate/severe impairment (based on standardized evaluations) or death
Chalak et al., 2021 [21]	USA (National Institute of Child Health and Human Development (NICHD) Neonatal Research Network)	Nested cohort study within an RCT	120	Asphyxiated neonates receiving hypothermia; mean gestational age 39 weeks	18–22 months	Composite result: either moderate or severe impairment or death
Sutin et al., 2023 [22]	USA (Boston)	Prospective, multicentre, observational cohort study	64 (73 received TH, 64 analyzed)	Perinatal asphyxia and probable HIE in newborns older than 34 weeks; HIE that is mild to moderate (median NE score 4)	18 months	BSID-III

All studies enrolled neonates with moderate-to-severe HIE treated with th, though severity distributions varied. For example, Mbatha et al. [15] reported 88% moderate and 10% severe HIE, while Jacob et al. [13] reported an 88.6% seizure rate and 72.7% coagulopathy. Follow-up ages ranged from 18 months to seven years. Most studies assessed outcomes at 18-24 months [14,15,18,21,22]; Glass et al. [17] followed children to 24 months, and Burgod et al. [20] to 18 months. One study extended follow‑up to 36 months [13], and Ancona et al. [19] reported the longest follow‑up at seven years.

Assessment instruments varied across studies: the Bayley Scales of Infant and Toddler Development (BSID; third or fourth edition) were used in five studies [14,16,17,18,22]; Jacob et al. [13] used the ASQ‑3; Mbatha et al. [15] used the Griffiths Mental Developmental Scales; and Ancona et al. [19] used intelligence quotient (IQ) testing and cerebral palsy diagnosis. Chalak et al. [21] and Burgod et al. [20] employed composite outcomes of death or disability.

In Table 2, favourable outcomes were reported across the majority of survivors, with substantial variability in proportions. Jacob et al. [13] reported that 79.5% of infants had normal neurodevelopment at 36 months, with early breastfeeding initiation and early hospital discharge identified as significant predictors (P=0.001). Veeraiah et al. [14] found that 81.2% had mild delay or normal development on BSID‑IV, while 18.8% had moderate‑to‑severe delay. Mbatha et al. [15] observed that the proportion of children with moderate‑to‑severe disability increased from 6% at 12 months to 32% at 18-24 months, underscoring the importance of extended surveillance.

**Table 2:** Neurodevelopmental outcomes and key findings

Study (Author, year (Reference))	Primary Outcome Measure	Key Neurodevelopmental Findings	Predictors/Associations	Mortality (If Reported)	Limitations/Notes
Jacob et al., 2025 [13]	Normal neurodevelopment at 36 months (Ages and Stages Questionnaire-3 (ASQ-3))	35/44 (79.5%) had normal neurodevelopment	Early breastfeeding initiation (< 48 hours) and early hospital discharge (< 48 hours after therapeutic hypothermia (TH)) predicted normal outcomes (P=0.001)	Not mentioned (NM)	Descriptive observational; outborn neonates; high seizure rate (88.6%)
Veeraiah et al., 2025 [14]	Moderate-to-severe developmental delay (The Bayley Scales of Infant and Toddler Development, Fourth Version (BSID-IV))	6/32 (18.8%) moderate-severe delay; 26/32 (81.2%) mild or normal	NM (but study supports TH use in low- and middle-income countries (LMICs))	NM	15 lost to follow-up (31.9%); limits generalizability
Mbatha et al., 2021 [15]	Moderate-to-severe disability at 18–24 months	At 18–24 months, 32% had moderate-to-severe impairment (compared to 6% at 12 months).	Sensitivity of 12-month assessment for 18-24 month outcome: 50%; specificity 100%	NM	High attrition (50% disability attrition at 18-24 months); retrospective; TH outside the neonatal intensive care unit (NICU) setting
Giesinger et al. 2022 [16]	Composite: death, CP, or Bayley-III <70	Adverse outcome predicted by tricuspid annular plane systolic excursion (TAPSE) <6 mm (91% sensitivity, 81% specificity)	TAPSE <6 mm (odds ratio (OR) 3.6) and abnormal brain MRI (OR 21.7) are independently associated with adverse outcome	9/46 (19.6%)	Small sample; physiological focus on right ventricular function
Glass et al. 2023 [17]	Death or neurodevelopmental impairment (NDI) at two years	Death or severe NDI are substantially correlated with severe EEG background abnormalities (adjusted relative risk (aRR) 7.95, 95% CI 3.49-18.12).	Severity of EEG background across the first 72 hours predicts outcome	Included in composite outcome (death or NDI)	EEG classification complex; trial population (erythropoietin vs placebo)
Boerger et al. 2024 [18]	Unfavorable neurodevelopmental outcome (death or BSID-III impairment) at 18–24 months	14/45 (31%) unfavorable outcome	Unfavorable outcomes are linked to higher beginning and maximum blood lactate levels (adjusted OR (aOR) 1.37 and 1.35, respectively)	Included in the unfavorable outcome group	Retrospective, single-center, small sample
Ancona et al. 2025 [19]	Adverse outcome: death, cerebral palsy (CP), or intelligence quotient (IQ) <70 at 7 years	11/40 (27.5%) adverse outcome; 29/40 (72.5%) favorable outcome	21 urinary metabolites identified (e.g., γ-butyrolactone, aldosterone) as discriminators; MRI positive predictive value (PPV) 67%, negative predictive value (NPV) 96%	Included in adverse outcomes	First study linking acute metabolome to seven-year outcome; small matched analysis
Burgod et al., 2024 [20]	Death or a moderate to severe disability at 18 months	Long birth depression group: 62.2% adverse outcome; Short depression: 35.4% (P<0.001)	Long birth depression is associated with more severe hypoxic-ischemic encephalopathy (HIE), higher mortality, and more brain MRI injury	Long depression: 47.5%; Short depression: 26.4%	Exploratory analysis; LMIC setting (South Asia)
Chalak et al. 2021 [21]	At 18–22 months, death or moderate–severe impairment	A higher risk of death or disability is associated with rewarming-related seizures (RR 1.7, 95% CI 1.25-2.37).	Electrographic seizures were more frequent during rewarming vs preceding epoch (27% vs 14% in group A)	Included in the composite outcome	Nested cohort; specific focus on rewarming period; good interrater agreement (κ=0.99)
Sutin et al., 2023 [22]	At 18 months, cognitive and motor composite scores (Bayley-III)	Cerebral metabolic rate of oxygen (CMRO₂) at normothermia was strongly correlated with both motor (β=2.77) and cognitive (β=4.49) scores; 21/22 (95%) had BSID-III scores >85.	When predicting outcomes, CMRO₂ performed better than NE score, cerebral fractional tissue oxygen extraction (cFTOE), and MRI/magnetic resonance spectroscopy (MRI/MRS).	One death (included in 22? Actually, one died out of 73, but final analysis N=22 with follow-up)	Small final sample (22 with complete data); high decline rate (33 declined); mild-moderate HIE only

Neurophysiological Markers

Glass et al. [17] demonstrated a strong association between severe EEG background abnormalities during hypothermia and death or severe neurodevelopmental impairment at two years (adjusted RR 7.95, 95% CI 3.49-18.12). Chalak et al. [21] reported that electrographic seizures during rewarming were more frequent than in the preceding epoch and were associated with a 1.7‑fold increased risk of death or disability at 18-22 months (95% CI 1.25-2.37).

Biochemical and Perinatal Markers

Boerger et al. [18] found that higher initial and maximal blood lactate levels during TH were independently associated with unfavourable outcomes at 18-24 months (adjusted OR 1.37 and 1.35, respectively). Burgod et al. [20] reported that prolonged birth depression (positive pressure ventilation >10 minutes or Apgar <6 at 10 minutes) was linked to higher mortality (47.5% vs. 26.4%) and greater death or disability at 18 months (62.2% vs. 35.4%).

Imaging, Cardiac, and Metabolic Markers

Giesinger et al. [16] identified that tricuspid annulus plane systolic excursion (TAPSE) <6 mm at 24 hours predicted adverse outcome (death, cerebral palsy, or BSID‑III <70) with 91% sensitivity and 81% specificity. Ancona et al. [19] performed a pioneering metabolomic analysis, identifying 21 urinary metabolites (e.g., γ‑butyrolactone, aldosterone) that distinguished favourable from adverse outcomes at seven years; brain MRI demonstrated a positive predictive value of 67% and a negative predictive value of 96%. Sutin et al. [22] measured cerebral metabolic rate of oxygen (CMRO₂) using non‑invasive spectroscopy and found that CMRO₂ at normothermia positively correlated with cognitive and motor Bayley‑III scores at 18 months, outperforming conventional clinical assessments and MRI.

Mortality

Mortality was variably reported: Giesinger et al. [16] reported 19.6% (9/46); Burgod et al. [20] reported 47.5% in the prolonged depression group; and Ancona et al. [19] included death as part of their adverse outcome composite.

Table 3 assesses the risk of bias for the given studies. Although the data from this research are generally of moderate to high quality, readers should exercise care when interpreting the results of fair-quality studies, especially those that have poor confounding control or a significant loss to follow-up.

**Table 3:** Risk of bias assessment by study design and domain Q1: prognosis; Q2: prognostic factors; Q3: treatment effect

Study (Reference)	Design/Review Question	Tool Used	Key Biases Assessed (Domains)	Overall Judgement	Notes
Jacob et al., 2025 [13]	Descriptive cohort (Q1)	Joanna Briggs Institute (JBI) Checklist	Attrition: High; Outcome validity: Low; Representativeness: Moderate	High attrition concern	31.9% loss to follow‑up
Veeraiah et al., 2025 [14]	Prospective cohort (Q1)	JBI Checklist	Attrition: High; Outcome validity: Low; Confounding: Not addressed	High attrition concern	32% loss to follow‑up
Mbatha et al., 2021 [15]	Retrospective cohort (Q1)	JBI Checklist	Attrition: High (50%); Outcome validity: Low; Representativeness: Moderate	High attrition concern	50% disability attrition
Giesinger et al., 2022 [16]	Physiological study (Q1/Q2)	Quality in Prognosis Studies (QUIPS)	Attrition: Low; Outcome: Low; Confounding: Moderate; Statistical: Low	Low to moderate risk	Small sample size (N=46)
Glass et al., 2023 [17]	Nested RCT cohort (Q2/Q3)	Cochrane Risk of Bias 2 (RoB 2) (randomized controlled trial (RCT))	Randomisation: Low; Missing data: Moderate; Outcome: Low	Some concerns	Nested within the erythropoietin trial
Boerger et al., 2024 [18]	Retrospective cohort (Q2)	QUIPS	Attrition: Moderate; Confounding: Low; Measurement: Low	Moderate risk	Single centre, small sample
Ancona et al., 2025 [19]	Longitudinal cohort (Q2)	QUIPS	Attrition: Moderate (24.5%); Confounding: Low; Outcome: Low	Low to moderate risk	Novel metabolomics focus
Burgod et al., 2024 [20]	RCT exploratory analysis (Q3)	RoB 2 (RCT)	Randomisation: Low; Missing data: Moderate; Outcome: Low	Some concerns	HELIX trial analysis
Chalak et al., 2021 [21]	Nested RCT cohort (Q3)	RoB 2 (RCT)	Randomisation: Low; Missing data: Low; Outcome: Low	Low risk	National Institute of Child Health and Human Development (NICHD) trial nested cohort
Sutin et al., 2023 [22]	Prospective cohort (Q2)	QUIPS	Attrition: High (decline rate 33%); Confounding: Low; Measurement: Low	High attrition concern	Only 22/73 analysed

Discussion

For most survivors, TH is associated with positive long-term neurodevelopmental results. A total of 1,134 infants with moderate-to-severe HIE treated with TH were included in this systematic evaluation of 10 trials. It also identifies several important predictors of unfavorable outcomes [13-22]. These predictors included delayed cooling initiation, elevated blood lactate, severe EEG abnormalities, rewarming seizures, abnormal brain MRI, and prolonged birth depression. However, their clinical utility warrants careful debate: most derive from single‑arm cooled cohorts without normothermic comparators, meaning they represent associations rather than validated prognostic tools. Furthermore, the timing, standardisation, and inter‑rater reliability of these measures varied considerably across studies, raising questions about their applicability in routine practice. Notably, while abnormal brain MRI consistently showed high negative predictive value, its positive predictive value was modest, and novel biomarkers such as TAPSE and urinary metabolomics, though promising, remain insufficiently validated for clinical adoption. These limitations underscore the need for prospective validation of multimodal prediction models before translating these predictors into routine prognostication. The included studies consistently reported normal or mildly abnormal neurodevelopment in 52%-81% of children followed to 18-36 months of age [13,14,17,22], and mortality rates ranged from 3.8% to 19.6% across studies [16-18]. These results are in line with the seminal RCTs that over the past 20 years have made TH the most effective treatment for moderate-to-severe HIE. The primary composite outcome of mortality or moderate-to-severe disability at 18-22 months dropped from 62% to 44% after cooling to 33.5°C for 72 hours (risk ratio, 0.72; 95% CI, 0.54-0.95; P=0.01), according to the NICHD whole-body hypothermia study (2005) [23]. In babies with less severe baseline aEEG abnormalities, the CoolCap trial (2005), which used targeted head cooling, found that the intervention enhanced survival without substantial neurodevelopmental damage (OR, 0.42; 95% CI, 0.22-0.80; P=0.009) [24]. Although there was no statistically significant difference in the key composite outcome of death or severe disability (RR, 0.86; 95% CI, 0.68-1.07; P=0.17), improved neurologic outcomes among survivors, such as decreased chances of cerebral palsy and higher Bayley Mental and Psychomotor Developmental Index scores, were verified by the following TOBY study (2009) [25]. 

Since then, a significant amount of meta-analytic research has supported these conclusions. Cooling lowers mortality (mean risk ratio, 0.75; 95% CI, 0.64-0.88) and enhances survival without significant neurodevelopmental impairment (typical risk ratio, 1.63; 95% CI, 1.36-1.95) without increasing major disability in survivors, according to Jacobs and colleagues' Cochrane review, which included eight RCTs involving 1,205 infants [26]. An updated meta-analysis by Tagin and colleagues that included seven trials with 1,214 babies found a substantial reduction in the risk of mortality or serious neurodevelopmental impairment at age 18 months (RR, 0.76; 95% CI, 0.69-0.84). Whole-body and selective head cooling methods were consistently beneficial for both moderate and severe HIE [27]. A pooled composite outcome (mortality plus severe impairment) of 39.2% (95% CI: 32.7%-46.1%) following TH was reported in a 2026 meta-analysis of 11 trials including 1,466 neonates. Whole-body cooling proved to be more effective than selective head cooling (RR = 0.71, P = 0.03) [8]. The robustness of TH as a neuroprotective intervention is supported by the consistency between our included studies and the larger body of research, with the number required to treat estimates ranging from seven to 11 to prevent one death or serious impairment [26,27].

The high correlation between early clinical and physiological indicators and long-term neurodevelopmental outcomes is a particularly noteworthy conclusion across several studies in this analysis. Early TH introduction during the first two to six hours of birth, lower initial and maximum blood lactate levels, the lack of significant EEG background abnormalities, and normal brain MRI findings were all independently linked to favorable outcomes, according to many included studies [13,17-19,21]. According to Glass et al., children with substantial EEG background abnormalities at any point during hypothermia were nearly eight times more likely to die or have severe neurodevelopmental damage at two years of age (adjusted RR: 7.95, 95% CI: 3.49-18.12) [17]. This conclusion is consistent with a large multicenter study that demonstrated a negative predictive value of 92% for normal or slightly abnormal outcomes when there were no seizures, whereas an 88% positive predictive value for mortality or serious impairment was found in a significantly aberrant aEEG at 48 hours of life [28]. The best predictive accuracy is achieved by combining an MRI at term-equivalent age with an aEEG during the first 72 hours. A thorough analysis of EEG and MRI characteristics for predicting long-term neurological outcomes in cooled newborns found that aberrant background patterns were the greatest predictors [29].

Brain MRI has similarly emerged as a powerful prognostic tool in the era of TH. A 2024 systematic review of 1,505 infants treated with TH found that MRI abnormalities, particularly those involving the basal ganglia and thalami, strongly predicted subsequent neurodevelopmental impairment, with pooled sensitivity and specificity values exceeding 80% for adverse outcomes [30]. According to Ancona et al., at seven years, brain MRI revealed a negative predictive value of 96% and a positive predictive value of 67% for adverse outcomes. supporting the importance of early neuroimaging for long-term prognostication [19]. Furthermore, there was a significant correlation between elevated glucose and lactate levels at 24 hours of TH and a higher chance of an adverse outcome, based on research examining the glucose-to-lactate ratio in hypothermic newborns with HIE. The ORs for lactate and glucose were 1.92 (95% CI 1.03-3.60, P = 0.041) and 30.2 (95% CI 1.47-62.07, P = 0.027), respectively, correspondingly. However, the result was not independently predicted by the glucose-to-lactate ratio [31]. These predictors underscore the importance of multimodal prognostication incorporating clinical, biochemical, neurophysiological, and neuroimaging parameters to guide family counselling and early intervention planning.

Several studies in this review identified novel biomarkers and physiological parameters that may refine prognostic accuracy beyond traditional predictors. Giesinger et al. showed that right ventricular dysfunction measured by TAPSE <6 mm at 24 hours of age independently predicted adverse outcomes (death, cerebral palsy, or Bayley-III <70) with 91% sensitivity and 81% specificity, highlighting the previously underappreciated role of cardiovascular function in neurological recovery after HIE [16]. This finding is supported by physiological studies demonstrating that post-ischaemic myocardial dysfunction is common after perinatal asphyxia and may exacerbate secondary brain injury through impaired cerebral oxygen delivery [16]. Even after adjusting for Sarnat stage, Boerger et al. found that a worse outcome at 18-24 months was associated with higher start and maximal blood lactate levels during TH, with adjusted ORs of 1.37 (95% CI: 1.01-1.88) and 1.35 (95% CI: 1.01-1.81), respectively [18]. Elevated lactate is a direct marker of anaerobic metabolism and tissue hypoxia, and its persistence during cooling may indicate inadequate neuroprotection or more severe initial insult [18]. 21 metabolites were discovered by Ancona et al., including creatinine, γ-butyrolactone, N-acetyl-galactosamine/glucosamine, and aldosterone. It separated positive from negative outcomes at age seven, with consequences for neuromodulation and neuronal vulnerability. This is the first study to associate long-term neurodevelopmental effects with acute-phase urine metabolomics [19]. These findings open promising avenues for personalised prognostication and targeted neuroprotective strategies, though they require validation in larger prospective cohorts.

Additionally, after adjusting for baseline clinical encephalopathy, seizures, and center, Chalak et al. showed that electrographic seizures during the rewarming phase were significantly more frequent than in the previous epoch and were linked to a 1.7- 95% CI: 1.25-2.37 times higher risk of mortality or disability at 18-22 months [21]. This highlights the need for ongoing EEG monitoring during the rewarming phase; the American Clinical Neurophysiology Society has amended its clinical recommendations to reflect this discovery, which now recommends extended monitoring through the rewarming phase given the increased seizure risk during this transitional period [32]. The mechanism may relate to altered cerebral metabolism and excitability as temperature normalises, potentially unmasking subclinical seizure activity [33].

An important emerging theme from both this review and recent literature concerns the trajectory of neurodevelopmental impairments beyond the traditional 18-24 month follow-up period. While many children appear to have normal outcomes at two years, longer-term follow-up studies reveal that new or worsening impairments can emerge or become apparent only at school age or adolescence. Jacob et al. followed children to 36 months and reported that 79.5% had normal neurodevelopment at that age [13], However, Mbatha et al. discovered that the sensitivity of evaluation at 12 months was only 50% for predicting 18-24 month outcomes, and the percentage of children with bad outcomes rose sharply from 6% at 12 months to 32% at 18-24 months [15]. Adverse outcomes rose from 14% at age two to 60% at age 6, subsequently somewhat dropped to 51% by age 10 in a long-term follow-up study of children receiving TH treatment. This suggests that even when early assessments are reassuring, subtle deficits in motor skills, cognition, and behavior may develop or worsen over time [34]. A 2023 population-based research of 66 babies treated with TH for HIE between 2007 and 2009 found that 17% of survivors experienced cerebral palsy, while the death rate was 12%, Research evaluation in early adolescence showed that 26% of kids with previously successful outcomes had developing deficiencies; between early school age and early adolescence, the proportion of kids with executive issues increased from 7% to 19% [35, 36].

Children with HIE treated with TH have been demonstrated to score significantly lower than typically developing children in the areas of fine motor skills, executive functions, memory, and language. They may also be less "school-ready" than their classmates who are typically developing [37]. According to a study of neurodevelopmental outcomes in a three-year-old Spanish cohort, 24% of survivors experienced negative outcomes, with 14% exhibiting minor neurological signs and more emotional problems than controls, such as introversion (10.5% vs. 1.3%), anxiety (34.2% vs. 11.7%), and depression (28.9% vs. 7.8%) [38]. These findings suggest that the full neurodevelopmental consequences of perinatal HIE may only become apparent when cognitive demands increase with age, and that current follow-up practices may underestimate the true burden of disability. Consequently, all children with a history of moderate-to-severe HIE, even those with normal early assessments, require long-term neurodevelopmental monitoring throughout adolescence. With regular assessments of executive function, behaviour, and academic achievement [39].

Over 90% of HIE infections happen annually in LMICs. The efficacy of TH remains an area of active debate and emerging evidence. Our review included several studies from LMICs that demonstrated encouraging outcomes. Veeraiah et al. in India reported that 81.2% of infants followed to 18-24 months had normal or mildly delayed development after TH, and Mbatha et al. in South Africa found that 68% of survivors had no moderate-to-severe disability at the same age, with the authors coming to the conclusion that, even when administered outside of a tertiary newborn critical care unit, TH was practical and possibly advantageous. [14,15]. TH was linked to a decrease in bad outcomes from 62.2% to 35.4% in the brief depression group compared to 35.4% in the long birth depression group, according to Burgod et al.'s exploratory analysis of the HELIX study, which involved 408 newborns in Bangladesh, Sri Lanka, and India. [20]. The TH group had a reduced composite of death or disability at six months or later (RR 0.78, 95% CI 0.66-0.92), but there was no significant difference in the newborn mortality rate between infants who were cooled and those who were not (RR 0.92, 95% CI 0.76-1.13), A recent systematic review and meta-analysis of 11 RCTs carried out in low- and lower-middle-income nations (1,324 newborns) [40]. The ILCOR Neonatal Life Support Task Force's meta-analysis (18 RCTs, 717 infants) found that the effect on survival is still unknown and that benefit or harm for It was not possible to rule out the primary outcome of mortality or neurodevelopmental impairment at 18-24 months (RR 0.63; 95% CI 0.38-1.04; p = 0.07) [41].

However, TH exceeds the standard of care for subsequent developmental outcomes and improves developmental outcomes after six months; 11 studies from LMICs were examined in a systematic review and meta-analysis using a health equity perspective. It discovered substantial impact sizes for differences in late developmental outcomes across groups (d = 0.85; CI = 0.62, 1.07) and high, significant effect sizes for changes in developmental outcomes within groups (d = -2.07; CI = -0.77, -3.36) [42]. These discrepant findings likely reflect substantial heterogeneity in study quality, baseline health system resources, patient selection, cooling protocols, and loss to follow-up across LMIC settings. They also highlight the critical importance of context-adapted implementation strategies rather than simple extrapolation of HIC protocols. The ILCOR Task Force concluded that where hospitals can follow defined TH protocols and provide adequate intensive care and follow-up, TH may offer meaningful benefit for term infants, but its effectiveness depends heavily on local health system capacity, resources, and adherence to protocols [41]. Large-scale trials such as the ongoing HELIX trial continue to address these questions [20,43].

Limitations

It is necessary to recognize a number of significant restrictions. First, there was significant variation in research design across the 10 included studies, sample sizes (44 to 408), HIE severity distributions, assessment tools (Bayley-II/III/IV, ASQ-3, Griffiths scales), definitions of adverse outcomes, and follow-up durations (18 months to 7 years), which precluded a formal meta-analysis. Second, loss to follow-up was a major concern: Veeraiah et al. lost 31.9% of infants, Mbatha et al. had 50% disability attrition, and Sutin et al. had a high decline rate with only 22 of 73 cooled infants included in the final analysis [14,15,22]. Selection bias may be introduced by high attrition. Third, although TH is presently the norm for treatment in affluent regions, it is challenging to determine absolute therapeutic effectiveness because most trials lacked a contemporaneous normothermic reference group. Fourth, even if funnel plot studies in recent meta-analyses did not find substantial bias, publication bias is still a possibility. Fifth, there was limited reporting of socioeconomic and demographic factors that could confound outcomes independently of HIE. Sixth, generalisability from HICs to LMICs is uncertain given differences in perinatal care and follow-up infrastructure. Seventh, there may be detection bias since none of the included research used blinding of outcome assessors to initial HIE severity or cooling settings. Finally, the predominance of short-term follow-up (18-24 months) may underestimate the full spectrum of neurodevelopmental sequelae, as impairments can emerge at school age.

## Conclusions

In the included observational cohorts, 52-81% of cooled survivors with moderate‑to‑severe HIE achieved normal or mildly delayed development at 18-36 months. However, because most studies lacked normothermic comparators, these outcomes cannot be causally attributed to TH based on this review alone. Consistent predictors of adverse outcomes included delayed cooling, elevated lactate, severe EEG, rewarming seizures, abnormal MRI, and prolonged birth depression. Emergence of impairments at school age supports surveillance beyond 24 months. While external landmark RCTs established TH efficacy in high‑income settings, LMIC data showed variable outcomes, heavily dependent on local resources. As a descriptive systematic review without meta‑analysis, definitive treatment‑effect conclusions cannot be drawn. Future research should validate biomarkers, standardise outcomes, and develop context‑appropriate LMIC protocols within comparative study designs.

## Appendices

Appendix A

Full Search Strategy

PubMed/MEDLINE

(Search date: [insert date]; Filters: English, Humans, last 5 years)

text

( "hypoxic-ischemic encephalopathy"[MeSH Terms] OR "hypoxic ischemic encephalopathy"[Title/Abstract] OR "neonatal encephalopathy"[Title/Abstract] OR "perinatal asphyxia"[Title/Abstract] ) AND ( "therapeutic hypothermia"[MeSH Terms] OR "therapeutic hypothermia"[Title/Abstract] OR "cooling"[Title/Abstract] OR "whole-body cooling"[Title/Abstract] OR "selective head cooling"[Title/Abstract] ) AND ( "neurodevelopmental outcomes"[Title/Abstract] OR "Bayley"[Title/Abstract] OR "cognitive"[Title/Abstract] OR "motor"[Title/Abstract] OR "cerebral palsy"[Title/Abstract] OR "long-term follow-up"[Title/Abstract] )

Limits: English language, human studies, full text available, published January 2021 - December 2026.

Scopus

text

TITLE-ABS-KEY ( "hypoxic-ischemic encephalopathy" OR "neonatal encephalopathy" OR "perinatal asphyxia" ) AND TITLE-ABS-KEY ( "therapeutic hypothermia" OR "cooling" OR "whole-body cooling" OR "selective head cooling" ) AND TITLE-ABS-KEY ( "neurodevelopmental outcomes" OR "Bayley" OR "cognitive" OR "motor" OR "cerebral palsy" OR "long-term follow-up" )

Limits: Language = English; Document type = Article; Publication year = 2021-2026.

Web of Science

text

( TS = ("hypoxic-ischemic encephalopathy" OR "neonatal encephalopathy" OR "perinatal asphyxia") ) AND ( TS = ("therapeutic hypothermia" OR "cooling" OR "whole-body cooling" OR "selective head cooling") ) AND ( TS = ("neurodevelopmental outcomes" OR "Bayley" OR "cognitive" OR "motor" OR "cerebral palsy" OR "long-term follow-up") )

Limits: Language = English; Document type = Article; Publication year = 2021-2026.

CENTRAL

text

#1 MeSH descriptor: [Hypoxic-Ischemic Encephalopathy] explode all trees #2 hypoxic-ischemic encephalopathy OR neonatal encephalopathy OR perinatal asphyxia #3 #1 OR #2 #4 MeSH descriptor: [Hypothermia, Induced] explode all trees #5 therapeutic hypothermia OR cooling OR whole-body cooling OR selective head cooling #6 #4 OR #5 #7 #3 AND #6 #8 neurodevelopmental outcomes OR Bayley OR cognitive OR motor OR cerebral palsy OR long-term follow-up #9 #7 AND #8 #10 #9 with publication year from 2021 to 2026
